# Monkey see, monkey do? Exploring parent-athlete behaviours from youth athletes' perspective

**DOI:** 10.3389/fspor.2023.1292812

**Published:** 2024-01-03

**Authors:** Liam P. McCabe, Margarita D. Tsiros, Alyson J. Crozier

**Affiliations:** ^1^ UniSA Allied Health and Human Performance, University of South Australia, Adelaide, SA, Australia; ^2^ Innovation, Implementation and Clinical Translation in Health, University of South Australia, Adelaide, SA, Australia; ^3^ Alliance for Research in Exercise, Nutrition and Activity, University of South Australia, Adelaide, SA, Australia

**Keywords:** psychology, team sport, prosocial behaviour, antisocial behaviour, social influence

## Abstract

Parents are an important social agent that can shape their child's behaviour in sport. However, the association between a youth athlete's perception of their parent's sideline sport behaviour and their own sporting behaviours is currently unclear. Therefore, the purpose of the present study was to explore the relationship between parent and youth athlete behaviours in sport settings. Australian youth athletes (*n *= 67) participating in team-based sports completed an online survey where they reported their parents positive and negative sideline behaviours and their own prosocial and antisocial sport behaviour during the past month. Linear regression results suggested that parent's positive behaviours were associated with youth prosocial behaviours, whereas parent's negative behaviours were associated with youth antisocial behaviours. Results provide preliminary quantitative evidence that youth athletes' perceptions of their parents' sideline behaviours predict their own on-field behaviours. As antisocial athlete behaviours were positively associated with parent negative behaviours, sport organisations should target, and ideally eliminate, negative parent behaviours. Conversely, to improve prosocial athlete behaviour, encouraging positive parent behaviours should be promoted.

## Highlights

• Positive sideline parent behaviours were frequently perceived, whereas negative sideline parent behaviours were rarely perceived by youth athletes
• Positive sideline parent behaviours were linked with more prosocial youth behaviours towards both their teammates and opponents
• Negative sideline parent behaviours were linked with more antisocial youth behaviours towards both their teammates and opponents

## Introduction

Sport participation promotes positive youth development opportunities, including prosocial values in young athletes, such as good sportsmanship and helping others when they are hurt (1). However, sport participation may also potentially foster antisocial values, such as cheating and a win-at-all costs mentality (2). Whether an athlete develops prosocial and/or antisocial morals in sport may be partially dependent on the social environment shaping youth experiences within sport.

Indeed, the youth sport environment can be shaped by a variety of social agents, with most attention paid to the coaches' and peers' influence (3). However, parents also play an essential part in the youth sporting experience (4), and may influence youth behaviour through processes such as modelling (5), reinforcement (6), and collaboration (7).

Parents modelling appropriate behaviour to their children aligns with the premise of Social Learning Theory (8), such that youth will learn what behaviour is appropriate by observing their parents' behaviours and associated consequences. Indeed, qualitative research has identified the presence of positive and negative parent behaviours, as well as a proposed link between parents and their children's sport behaviour (9, 10). Quantitatively, research that has observed parent's behaviours (using an external observer) found that positive and negative parent behaviours were significant correlates of positive and negative youth athlete behaviour (11–15). While the use of external observers provides an objective behavioural assessment, it was challenging for observers to capture *all* spectator behaviours (11). Researchers have also explored the parent-youth athlete behaviour relationship by asking parents to self-report their own behaviours (16, 17). Parents generally reported high frequencies of positive behaviour and low frequencies of negative behaviour, and a significant relationship between parent-youth athlete behaviours was found. However, what is missing from the parent-youth athlete behaviour literature is youth athlete's voice, and whether their *own perception* of their parents' sideline behaviour relates to their own on-field behaviour. As one exception, research by Dorsch and colleagues (18) explored youth perceptions of the frequency of some parent behaviours – youth perceived a high frequency of support and warmth and a low frequency of conflict and pressure behaviours from their parents in the sport context. However, this research was not specific to the sport sideline behaviours (e.g., cheering or yelling during a game) and did not examine the relationship between parent and youth behaviours.

The current pilot investigation explores relationships between parent and athlete behaviours in sport from youth athletes' perspectives. Grounded in Social Learning Theory (8), it was hypothesized that positive parent behaviours would be positively associated with prosocial youth athlete behaviour, and negative parent behaviours would be positively associated with antisocial youth athlete behaviour.

## Methods

### Participants

Participants included Australian youth athletes (12–17 years) who played team-based sport (e.g., soccer, basketball, rugby) with a parent/primary caregiver in attendance during the previous month. Youth who had not played sport over the last month, had an injury in the preceding month impacting their sport participation, had a physical/intellectual disability, or played an individual-based sport (e.g., swimming, golf) were excluded from participation.

### Design and methodology

Ethics approval was provided by the university's Human Research Ethics Committee (protocol number 203229). After ethics approval, the survey was piloted for interpretability with two youth, aged 12 and 17 years from a single sports club prior to wider dissemination. No changes were made to the survey, as feedback indicated the survey was interpretable and easy to understand.

Parents of youth athletes were recruited from October 2020-March 2021, through paid social media advertisements and flyers/emails distributed through sport clubs (i.e., convenience sampling). Parents completed an online screening questionnaire to determine youth eligibility, viewed an information sheet and indicated their consent before being emailed a personalised youth survey link. The use of the personalised survey link allowed researchers to verify parental informed consent with youth participant survey responses, which is a feature available in the REDCap survey software used (v10.0.19, Vanderbilt University, Nashville, TN, USA). Youth participants viewed an information sheet and provided assent to participate.

Data collection occurred in states/territories that were not experiencing social distancing restrictions due to COVID-19 outbreaks within Australia (such that youth sport including spectators was occurring). Youth completed the survey at a convenient time, in a self-selected location with internet/computer access. Youth were instructed to complete the questionnaire independently, without influence from others, including their parents, siblings, or friends. The survey first asked questions surrounding participant demographics, including age, gender, state of residence, and sport details (i.e., the sport(s) they currently played, duration of sport participation, and level of sport played). Participants were then asked to reflect on their last month of sport participation and report their perceptions of their parents, as well as their own, positive and negative behaviours within the sport setting.

### Measures

Whereas youth prosocial and antisocial behaviour has a well-used valid and reliable measurement tool, a search of the literature did not identify a valid and reliable measure for assessing parent sideline behaviours. As such, purpose-built scales were developed to measure negative and positive parent behaviour. Examples of positive behaviours included cheering, encouraging athletes, and helpful comments around supporting and assisting others off the floor or if injured. Examples of negative behaviours included yelling, swearing, put-downs, getting annoyed, intent to hurt/threaten/injure/foul/distract/get revenge on others, reacting badly to a loss/foul or breaking rules. For this study, youth were asked to reflect on the frequency of specific behaviours (in relation to themselves and their parent) and not whether they perceived them to be positive or negative behaviours *per se*.

#### Negative parent behaviour

Five items assessed negative parent behaviours modified from a questionnaire by Shields et al. (16). For the current study, the items were modified to examine *youth perceptions* of their parents' negative sport behaviours (e.g., “During the last month, how often has your parent encouraged you to hurt a player on the other team?”). For all five-items, participants reported the frequency that their parents engaged in each behaviour while attending their sporting events over the previous month, using a 5-point Likert type scale, ranging from (1) *never* to (5) *very often.* Internal consistency for the five items was marginally below acceptable levels [Cronbach's alpha = 0.62; (19)]. Removal of any one item did not improve consistency; thus, all five items were aggregated and averaged.

#### Positive parental behaviour

Four items were developed to assess positive parent behaviour, guided by the Parent Observation Instrument for Sport Events [POISE; (12)]. Participants reported the frequency that their parents engaged in each of four positive behaviours (e.g., “During the last month, how often has your parent cheered for you during the game?”), using a 5-point Likert type scale, ranging from (1) *never* to (5) *very often.* Internal reliability for the positive items was considered acceptable [Cronbach alpha = 0.75; (19)]. Thus, the four positive items were aggregated and averaged for data analysis.

#### Youth self-reported behaviours

To assess youth sport behaviours, the Prosocial and Antisocial Behaviour in Sport Scale (PABSS) was administered, which consists of 20 questions assessing athletes' perceived frequency of engagement in positive and negative behaviours in sport (20). The PABSS was developed for use with team-based sports and assesses prosocial and antisocial youth behaviours towards teammates and opponents, resulting in four subscales: Prosocial-Teammate, Prosocial-Opponent, Antisocial-Teammate, and Antisocial-Opponent. The PABSS is responded to on a 5-point Likert scale, ranging from (1) *never* to (5) *very often*, with items for the subscales aggregated and averaged, as per scoring protocols (21). Questions assessing prosocial and antisocial behaviours were interspersed to reduce potential reporting bias. Further, to limit recall bias, participants only reflected on behaviour experienced within the last month. A one-month recall period is conservative compared with previous research using the PABSS that has required youth to reflect on their behaviours “*throughout the season*” at 2 weeks, 6–8 weeks and at 12–16 weeks into their sporting season (22).

The PABSS has established concurrent, construct, content and discriminate validity in youth team sport settings (20). Acceptable test-retest reliability (ICC .75–.95) and internal consistency (range = .74–.86) has also been established in youth athletes (23). Internal consistency for the current study were deemed acceptable, ranging from .69 (Prosocial-Opponent) to .85 (Prosocial-Teammate) (19).

## Data analysis

All survey data responses were imported to SPSS (V.25, International Business Machines Corporation, New York, NY, USA) and examined for normality, outliers and missing data. Only one variable was normally distributed (Prosocial-Opponent) and was not transformed. Logarithmic transformations were performed for negative parent behaviour, youth antisocial-opponent behaviour and positive parent behaviour (the latter was also reflected). Square root transformations were applied to youth antisocial-teammate and prosocial-teammate behaviours (the latter was also reflected). As different transformations were used, z-scores were calculated to standardize the means/standard deviations for the main analyses to aid interpretability. Little's MCAR test was nonsignificant (*p* = .99), indicating that the seven missing data points were missing at random. Expectation maximisation imputation was then used to impute data based on participants' other responses within that scale (24). Age was positively correlated with positive parent behaviour (*r *= 0.27, *p* < .05), and was thus controlled for in subsequent analyses.

To examine whether positive and negative parent behaviour (independent variables) were associated with youth athlete behaviour, four linear regressions were conducted with each of the dependent variables assessing athlete behaviour. Significance was set at *p* = 0.05. An *a priori* power calculation indicated a sample size of 47 would detect a correlation of 0.4 (medium effect size) at an alpha level of 0.05, with 80% power.

## Results

### Participants

A total of 197 parents accessed the survey/information sheet, with 195 providing consent for their child to participate. Sixteen potential participants did not meet an inclusion criterion (no sport played in the last month, *n* = 1; parent did not attend their sport within the last month, *n* = 2; outside age range of interest, *n* = 2; physical/intellectual disability reported, *n* = 6; played individual-based sport, *n* = 2; or reported not completing the survey independently, *n* = 3), and thus were excluded. Therefore, 183 customised survey links were sent to potential youth participants. Of those 179, 108 did not access the survey, 4 started the survey but did not complete enough questions to be able to look at relationships, and 67 provided assent and completed the survey with sufficient data for inclusion (37.4% retention rate).

Descriptive statistics for participant characteristics are presented in Table 1. Overall, 67 youth athletes with an average age of 13.8 years (*SD* = 1.4) participated in the study. Most participants identified as male (*n* = 36, 53.7%), with the rest identifying as female (*n* = 31, 46.3%). Participants came from all states and one territory in Australia; however, the majority came from South Australia (37.3%), Queensland (20.9%), and New South Wales (17.9%). Most youth had been playing sport for at least four years (76.2%) and played sport at the club level (64.2%). A variety of sports were reported (*n* = 11), although the most prevalent sports played by participants were soccer (28.4%) and netball (17.9%).

**Table 1:** Participant demographic descriptive statistics.

Demographic variables	Categories	*n* = 67
Age (years)		*M* = 13.87 ± 1.42
Gender	Male	36 (53.7%)
	Female	31 (46.3%)
State	ACT	2 (3.0%)
	New South Wales	12 (17.9%)
	Queensland	14 (20.9%)
	South Australia	25 (37.3%)
	Tasmania	2 (3.0%)
	Victoria	7 (10.4%)
	Western Australia	5 (7.5%)
Level of sport	School	6 (9.0%)
	Club	43 (64.2%)
	State	15 (22.4%)
	National	3 (4.5%)
Sport experience	1–2 years	5 (7.5%)
	4–8 years	33 (49.3%)
	>8 years	18 (26.9%)
	2–4 years	11 (16.4%)
Sport most played	Australian Rules Football	2 (3.0%)
	Baseball	5 (7.5%)
	Basketball	7 (10.4%)
	Cricket	9 (13.4%)
	Hockey (Field)	3 (4.5%)
	Lacrosse	1 (1.5%)
	Netball	12 (17.9%)
	Rugby	6 (9.0%)
	Soccer	19 (28.4%)
	Water polo	2 (3.0%)
	Other	1 (1.5%)

### Descriptive statistics

**Figure 1:**
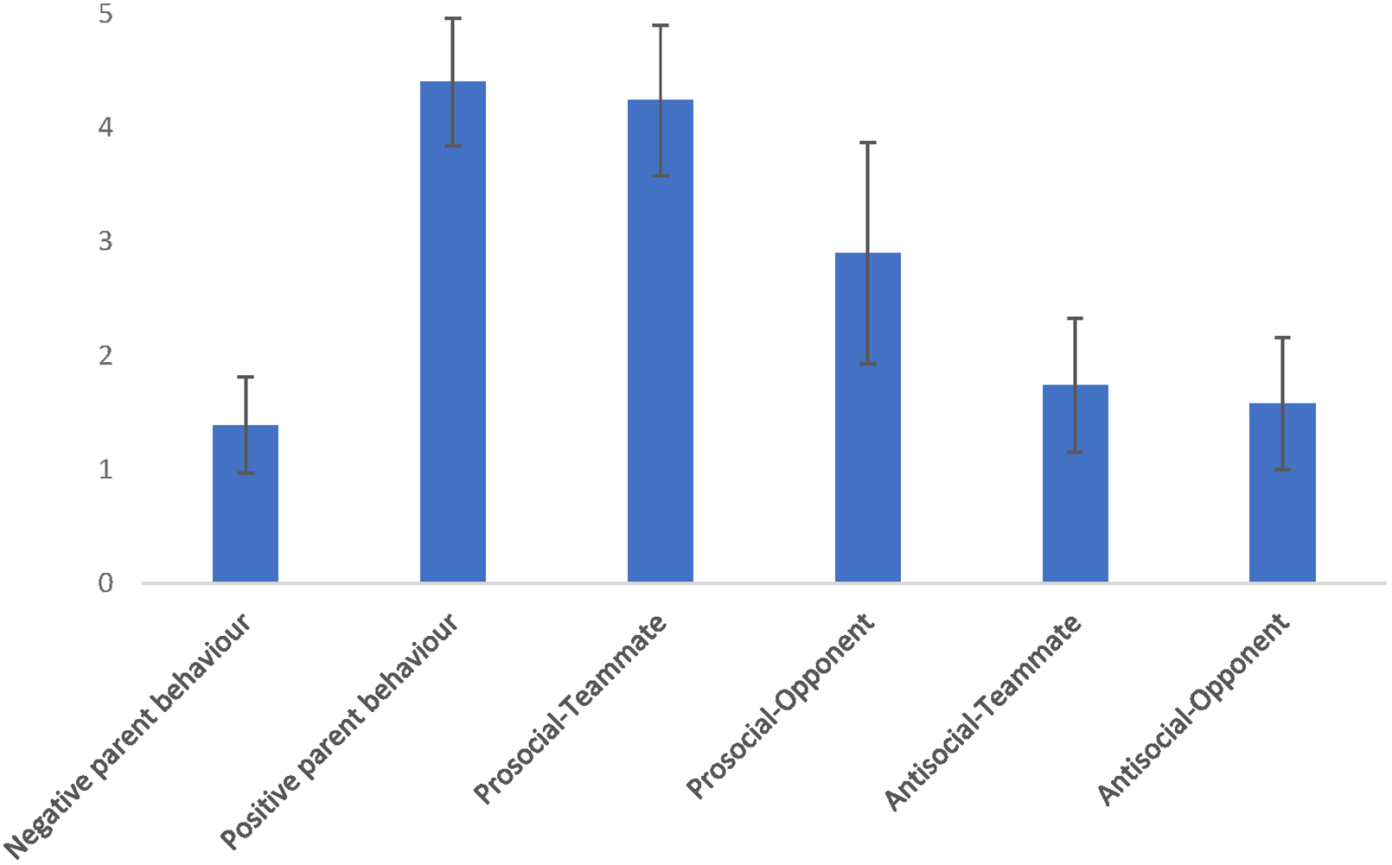
Parent and youth sport behaviours. Data are presented as means and standard deviations assessed on 5 point Likert scales.

Negative parent behaviour was considered low (*M* = 1.39, *SD* = .42) and positive parent behaviour was frequent (*M* = 4.40, *SD* = .56; see Figure 1). Youth reported frequently demonstrating Prosocial-Teammate behaviour (*M *= 4.24, *SD* = .66), whereas Prosocial-Opponent behaviour was sometimes reported (*M* = 2.90, *SD* = .97). Youth also reported rarely demonstrating both Antisocial-Teammate behaviour (*M *= 1.74, *SD* = .59) and Antisocial-Opponent behaviour (*M* = 1.58, *SD* = .58).

### Perceived parent-youth athlete behaviour relationship

The four linear regression analyses indicated that perceived parent behaviours were significantly associated with self-reported youth athlete behaviour (*p*'s < 0.05), accounting for 12.8%–35.4% of the variance in youth athlete behaviour. Age was not a significant predictor in all models, with positive parental behaviours associated with athlete prosocial behaviours (towards teammates and opponents) and negative parent behaviours associated with athlete antisocial behaviours (towards teammates and opponents). See Table 2 for more details.

**Table 2:** Relationship between parent and youth behaviours (*n* = 67).

Dependent variable	Predictor	*R* ^2^	*F*	*β*	*p*-value	*η* ^2^
Prosocial-Teammate		.**26**	**7**.**37**	** **	**<**.**001**	** **
	Age			-.08	.43	-.08
	Positive parent behaviour			.**47**	**<**.**001**	.**45**
	Negative parent behaviour			.22	.06	.21
Antisocial-Teammate		.**12**	**3**.**07**	** **	.**03**	** **
	Age			-.06	.57	-.06
	Positive parent behaviour			-.09	.46	-.08
	Negative parent behaviour			.**32**	.**01**	.**33**
Prosocial-Opponent		.**20**	**5**.**38**	** **	.**001**	** **
	Age			.09	.38	.09
	Positive parent behaviour			.**47**	**<**.**001**	.**45**
	Negative parent behaviour			-.02	.86	-.02
Antisocial-Opponent		.**24**	**6**.**72**	** **	.**001**	** **
	Age			.11	.30	.11
	Positive parent behaviour			-.08	.51	-.07
	Negative parent behaviour			.**43**	**<**.**001**	.**42**

Significant relationships in bold. Direction of the standardized beta values have been adjusted (where required due to transformations) for accurate interpretation.

## Discussion

The main purpose of this pilot study was to explore the relationships between parent-youth athlete behaviours in sport settings. Encouragingly, youth perceived their parents engaging in positive behaviours (e.g., cheering/clapping) relatively frequently. These positive parental behaviours were then a significant predictor of youth engagement in prosocial behaviours. Results also indicated that youth athletes perceived negative parent behaviours (e.g., being critical or encouraging youth to hurt the opposition) as rare. Despite being rare, a significant relationship was identified between negative parent behaviours and antisocial athlete behaviours.

Aligning with previous research exploring parent's self-reported positive behaviours (16, 17), the current study suggests athletes perceive a similarly high prevalence of positive parent behaviours in sport. Given that positive parent behaviour in sport has been identified to improve youth self-esteem (25), life skill development (26), and reduce anxiety (27), this finding is promising, and we should be encouraging parent positive behaviours to continue. Indeed, research has shown that youth found more enjoyment in their sporting experience when there was active and positive parental involvement in the form of encouragement from the sidelines (28).

Youth athletes also perceived low levels of negative parent behaviours, such as being critical or encouraging youth to hurt an opposition player. These findings are comparable with previous research whereby parents self-reported engaging in relatively low frequencies of negative behaviours during their child's sporting events (16, 29). While having low levels of perceived negative parent behaviours is encouraging, previous research that has *observed* parent behaviours reported an equal prevalence of positive and negative parent behaviours (14). As such, it may be that youth (in the current study) and parents [in prior research (16)] under-reported the prevalence of negative parent behaviours. The presence of any negative behaviours is still of concern. Poor parent communication and anger in sport has been identified to negatively impact youth well-being, emotionally and physiologically (17, 30). Negative parent behaviour is also associated with increased sadness, reduced confidence and a higher likelihood of youth withdrawing from sport (17). As such, the presence of negative parent behaviours, even if rare, could potentially lead to negative youth sport experiences and requires careful attention.

In line with our main hypothesis, there was a significant parent-youth athlete behaviour relationship found, which supports the premise of Social Learning Theory (8), such that through perceived parent behaviours, youth may learn what behaviour is acceptable. More specifically, results indicated that positive parent behaviour was positively related to youth prosocial behaviour in sport. This finding is consistent with previous research in ice hockey players, whereby youth perceptions of their parents' sport involvement (e.g., providing transport to/from practice and competitions) was associated with the amount of concern for, and level of graciousness toward, their opponents (31). Similarly, significant associations between positive parent and positive player behaviours in youth basketballers has been found when external observation of parent behaviours was conducted (11).

Also as expected, results showed that the more youth perceived their parents as engaging in negative behaviours, the more likely they reported also engaging in antisocial behaviours. Such findings are in line with previous research that found parents negative behaviours were associated with poor sportspersonship towards opponents (11, 32), as well as youth aggression and frustration during sport (17). As parents have been considered co-participants in the sport environment (33), the results of the current study provide preliminary evidence that the presence of negative parent behaviours may be associated with the presence of youth athlete antisocial behaviours.

Though not the focus of the current study, our results identified a positive correlation between positive parent behaviour and participant age. Older participants rated their parents as engaging in more positive behaviours than younger participants. It may be speculated that parents experience on the sport sidelines over time has modified their behaviours to be more encouraging in nature. Parents may perceive less stress associated with their child's sport engagement, as the child takes more ownership of their experience (e.g., driving themselves to and from practice) or the pressure to make the elite level may have changed. Although age was not associated with athlete self-reported behaviour in the main analyses, exploring athlete age in relation to parent sideline behaviours may be an avenue worthy of further research.

In addition, a result worth discussing was the lack of an association between positive parent behaviours and youth antisocial behaviours. This contrasts with a previous meta-analysis which found the presence of a sport environment that promoted prosocial behaviours was associated with reduced antisocial youth behaviours in sport (34). Researchers have also shown that when a higher parental or coach mastery motivational climate (i.e., a focus on personal improvement and exerting effort) is present, there is reduced youth antisocial behaviours towards teammates (32). Notably, prior research has examined a variety of social agents (including the coach), rather than parents alone (as done here), which could explain our differing findings. It may be that other social agents influence the youth athlete antisocial behaviour differently than parents. Given that both parents and coaches are thought to be important influences on youth athletes (6, 35), future research may be warranted to explore youth perceptions of their parents' and coaches' behaviours concurrently.

### Strengths & limitations

This study adds to the limited research exploring youth perspectives of parent's sport sideline behaviours, and the relationship between parent-youth athlete behaviour. Though prior research using external observers found a parent-youth athlete relationship existed (11, 15), this study was one of the first to demonstrate that an athlete's perception of their parents' behaviour was related to their own reported behaviour. We also took into consideration potential covariates in the regression equations, including participant age, sport type, and experience, which were found to be non-significant predictors of youth athlete behaviours. Further strengths of this study include the inclusion of a variety of team sports across Australia (potentially improving generalisability) and measuring youth athlete prosocial/antisocial behaviours with a frequently used and validated measure. Moderate effect sizes were found, which is what was expected from previous research, and thus the sample size was sufficient for the study purposes.

It is important to recognise several limitations in the current study. As this was a cross-sectional study, only associations between parent and youth behaviours in sport can be assumed. To determine causation, experimental studies are needed to determine whether modifying parent's sideline behaviours impacts their child's sport behaviours. Although this study assumed that parents would be the role model influencing youth behaviours, previous research has shown that when positive youth athlete experiences occur, parents behave in a positive manner, indicating that social learning is reciprocal (5). Thus, future research should explore the parent-child behaviour relationship in youth sport using longitudinal research designs to tease out directionality.

Moreover, as no valid or reliable measures for assessing parent behaviours were available, a purpose-built measure was developed based on prior measures (12, 16). Given that the internal consistency for the negative parent behaviour scale was slightly below acceptable levels, further research and psychometric testing is required to create a valid and reliable measure of parent sport behaviour. Further, participants were asked to reflect on overarching parent behaviour, but were not asked to specifically reflect on their mother's or father's behaviours (or both). Previous research has found that the gender of the parent may influence the relationship, with fathers exhibiting more negative behaviours than mothers during youth baseball and ice hockey (36). Future research should explore the relationships found in the current study separately for mothers and fathers.

As identified earlier, the current study purposefully examined only one of potentially many social agents who may be important in the youth sport context. Future research should investigate the multiple social agents which may impact youth athlete behaviour in sport simultaneously, to determine their independent and interactive effects. The low prevalence of negative behaviours reported for both parents and youth athletes may indicate that participants were likely to participate if undesirable behaviours were considered rare. Therefore, self-selection bias may have occurred at two levels: the parents and children. Due to social distancing restrictions with face-to-face data collection imposed by the university, the online sampling method was deemed most appropriate for the current study but impacted our ability to recruit a larger sample. As such, results should be interpreted with these limitations in mind. To confirm, replicating study findings across a broader demographic (which was limited by Covid-19 sport participation restrictions) would be valuable. Finally, although we conducted an *a priori* sample size calculation and were appropriately powered to detect a moderate relationship, our modest sample size may limit the generalizability of our study findings. We also cannot rule out the possibility of type I error, particularly in relation to smaller, less apparent effect sizes.

### Practical implications

If aiming to reduce antisocial behaviour and increase prosocial behaviour among youth athletes, the presence of positive parent behaviours should be encouraged, whereas negative parent behaviours should be discouraged, and ideally eliminated, within the sport environment. Specifically, to promote positive and reduce negative parent behaviours, sport organisations may wish to incorporate a sport parent behaviour guide and/or educative seminar. Researchers have shown that a parental “behaviour” guide and 45-minute seminar reported improvements in parental support, reduced pressure, and conflict (37). Similarly, the “Respect in Sport Parent Program” for adults is an online course designed to provide education around abuse, bullying, discrimination, and harassment to promote a safe, rewarding, and positive youth sport experience (https://www.respectgroupinc.com/respect-in-sport/#parent-program). The “Respect in Sport Parent Program” was found to be related to reduced youth antisocial behaviours and improved social skills (38). Overall, a systematic review identified that parent education programs and interventions had significant improvements on parents' and children's knowledge and skills, as well as parents sportspersonship behaviours (39). In junior Australian football, parents have also been required to sign an AFL Kids First policy “code of conduct” to encourage positive parental behaviour. This code of conduct has been shown to be effective leading into the season but has limited effectiveness to challenge parental behaviour throughout the season (40). Finally, to achieve effective change, an integrated approach that targets a variety of contexts where youth engage in sport (e.g., sporting organisations, schools, etc.) may be needed.

## Conclusion

Overall, the results of our study suggest that parents as spectators on the sport sidelines play an important role in shaping their child's behaviour. The findings from this study add to the limited previous research on youth perspectives of parent behaviour. The more youth perceived positive (or negative) parent behaviours, the more they reported engaging in comparable behaviours themselves towards their teammates and opponents. Sport organisations may wish to target, and ultimately eliminate negative parent behaviours, while promoting positive behaviours, thereby fostering positive youth experiences and on-field behaviours.

## Data Availability

The raw data supporting the conclusions of this article will be made available by the authors, without undue reservation.
